# Sex disparities in vitamin D status and the impact on systemic inflammation and survival in rectal cancer

**DOI:** 10.1186/s12885-021-08260-2

**Published:** 2021-05-11

**Authors:** Hanna Abrahamsson, Sebastian Meltzer, Vidar Nyløkken Hagen, Christin Johansen, Paula A. Bousquet, Kathrine Røe Redalen, Anne Hansen Ree

**Affiliations:** 1 Department of Oncology, Akershus University Hospital, Lørenskog, Norway; 2 Institute of Clinical Medicine, University of Oslo, Oslo, Norway; 3 Department of Multidisciplinary Laboratory Medicine and Medical Biochemistry, Akershus University Hospital, Lørenskog, Norway; 4 Department of Physics, Norwegian University of Science and Technology, Trondheim, Norway

**Keywords:** Rectal cancer, Vitamin D, Sex, Inflammation, Survival

## Abstract

**Background:**

We reported previously that rectal cancer patients given curative-intent chemotherapy, radiation, and surgery for non-metastatic disease had enhanced risk of metastatic progression and death if circulating levels of 25-hydroxyvitamin D [25(OH) D] were low. Here we investigated whether the association between the vitamin D status and prognosis pertains to the general, unselected population of rectal cancer patients.

**Methods:**

Serum 25(OH) D at the time of diagnosis was assessed in 129 patients, enrolled 2013–2017 and representing the entire range of rectal cancer stages, and analyzed with respect to season, sex, systemic inflammation, and survival.

**Results:**

In the population-based cohort residing at latitude 60°N, 25(OH) D varied according to season in men only, who were overrepresented among the vitamin D-deficient (< 50 nmol/L) patients. Consistent with our previous findings, the individuals presenting with T4 disease had significantly reduced 25(OH) D levels. Low vitamin D was associated with systemic inflammation, albeit with distinct modes of presentation. While men with low vitamin D showed circulating markers typical for the systemic inflammatory response (e.g.*,* elevated erythrocyte sedimentation rate), the corresponding female patients had elevated serum levels of interleukin-6 and the chemokine (C-X-C motif) ligand 7. Despite disparities in vitamin D status and the potential effects on disease attributes, significantly shortened cancer-specific survival was observed in vitamin D-deficient patients irrespective of sex.

**Conclusion:**

This unselected rectal cancer cohort confirmed the interconnection of low vitamin D, more advanced disease presentation, and poor survival, and further suggested it may be conditional on disparate modes of adverse systemic inflammation in men and women.

**Trial registration:**

ClinicalTrials.govNCT01816607; registration date: 22 March 2013.

**Supplementary Information:**

The online version contains supplementary material available at 10.1186/s12885-021-08260-2.

## Background

Evidence over the past decades supports that vitamin D has a beneficial effect on colorectal cancer (CRC) development and outcome [1–4]. The primary circulating form of vitamin D, 25-hydroxyvitamin D [25(OH) D], is mainly dependent on the exposure to ultraviolet B solar radiation, and levels are therefore depleted during the winter season at high latitudes unless the dietary supplementation is adequate [5]. It has been proposed that the association between the vitamin D status and CRC may not be causal but rather a marker of underlying inflammatory processes [6]. A wide range of molecular components, many of which are reflected in the systemic circulation, are involved in cancer-related inflammation [7]. The systemic inflammatory response (SIR) as such is an adverse prognostic marker in all stages of CRC [8, 9]. Furthermore, it is notable that both incidence and mortality rates of rectal cancer are significantly higher in men than in women [10, 11], which might relate to modifiable lifestyle factors with different impact in the two sexes [12].

In a study population of rectal cancer patients residing at latitude 58–62°N in Norway, which was recruited between 2005 and 2010 and given curative-intent chemotherapy, radiation, and surgery for locally advanced disease, we observed that serum 25(OH) D differed significantly over the seasons but was essentially similar for men and women [13]. The patients were at high risk of metastatic progression as almost 40% of cases presented with T4 stage and more than 85% had involved lymph nodes. Vitamin D deficiency, defined as 25(OH) D levels below 50 nmol/L [14–16], was predictive of the T4 disease stage, lack of tumor response to the neoadjuvant treatment, and significantly heightened risk of metastatic progression following the multimodal therapy, irrespective of the season of diagnosis. Only a weak inverse correlation was noted between serum 25(OH) D and common markers of systemic inflammation [13].

The locally advanced rectal cancer study was undertaken on a highly selected population of patients [17] who were planned for curative-intent neoadjuvant therapy and surgery, yet at high risk of metastatic progression beyond the pelvic cavity. The setting of non-metastatic but high-risk tumor stage is ideal to study biological processes involved in metastatic dissemination since a certain percentage of patients will proceed to metastatic disease even after curative-intent therapy. However, our previous findings on vitamin D and rectal cancer [13] may not necessarily be representative for the general, unselected population of patients, where the interconnection of the vitamin D status and systemic inflammation and the impact on prognosis in males compared to females may be disparate. Here we report on 129 rectal cancer patients enrolled onto a prospective population-based biomarker study. The study participants, residing at latitude 60°N in Norway, represented the entire range of disease stages and were recruited between 2013 and 2017. We examined how the patients’ circulating levels of 25(OH) D related to the season of diagnosis, male or female sex, inflammatory processes, and survival.

## Methods

### Study population

In this post hoc analysis, we used data from a prospective biomarker study in rectal cancer undertaken at Akershus University Hospital (Lørenskog, Norway). The hospital has a catchment area at latitude 60°N that covers a tenth of the Norwegian population and reflects essential demographics (age groups, socio-economic distribution, and ethnic composition) of the nation. Patients were enrolled according to an all-comers design (unselected patient recruitment within the program for standardized cancer diagnostic pathways in the public health services) between 28 October 2013 and 31 October 2017. Routine and research blood samples were collected at the time of inclusion. Patients had diagnostic staging of the disease described by the TNM classification system [18] with the tumor extension within the pelvic cavity determined by magnetic resonance imaging and the metastatic status determined by computed tomography of the thoracic and abdominal cavities. For the current analyses, disease stage was translated to the American College of Radiology (ACR) Appropriateness Criteria [19]. The patient cohort presented in this investigation (*n* = 129), stemming from 192 enrolled study patients, excluded cases that were screening failures (*n* = 6) or concluded with non-invasive adenoma (*n* = 11) or non-adenocarcinoma malignancy (*n* = 9) and patients who have withdrawn the study consent (*n* = 4) or are devoid of remaining serum for 25(OH) D analysis (*n* = 33).

### Clinical end point

Patients were treated according to the national guidelines with curative-intent surgery with or without neoadjuvant treatment (radiation with or without concomitant chemotherapy) or adjuvant chemotherapy, as appropriate, or palliative chemotherapy; none received an immune checkpoint inhibitor. The median follow-up time was 39 months (minimum, 3; maximum, 74) when censored on 2 January 2020. At this time, almost a third of patients had follow-up of more than 5 years. The primary clinical outcome variable in this post hoc analysis was cancer-specific survival (CSS), defined as the time from the date of study inclusion (2–3 days prior to histological confirmation of rectal adenocarcinoma) until death due to the rectal cancer.

### Measurement of 25(OH) D

Blood was drawn in plain serum tubes for centrifugation to separate serum, which were left on ice for no more than 1 h before storage at −80 °C. The content of 25(OH) D was measured with high-pressure liquid chromatography with ultraviolet detection at a limit of 10 nmol/L. The mean recovery was > 85% with an intra-assay coefficient of variation of 2.3% (*n* = 8) and an inter-assay coefficient of variation of 7.4% (*n* = 22).

### Identification of serum inflammation factors

The simultaneous analysis of 87 proteins broadly related to inflammation was undertaken with customized Luminex Multiplex Assays (R&D Systems), according to the manufacturer’s instructions. From these, 56 proteins were considered feasible for quantitative statistical analyses with the Significance Analysis for Microarrays (SAM) method [20] developed for multiple testing of associations. Because SAM imputes missing data values (via the K-Nearest Neighbor algorithm), it was only used for exploration of proteins possibly associated with 25(OH) D.

### Statistical analysis

All statistical analyses were performed in STATA 16. Patient demographics were described by mean and standard deviation (SD) or mean with minimum and maximum values for continuous variables, and frequency and percentage for categorical variables. Differences between groups were assessed by independent samples *t*-test, one-way analysis of variance, or chi-square test, as appropriate. When stratified by sex, associations between serum 25(OH) D values and patient and tumor characteristics were assessed by linear regression models to enable adjustment for season collapsed into winter/spring and summer/fall to avoid violation by small sample sizes. Because the adjustment did not substantially alter the results, adjusted *p*-values were left out. The correlation analysis of serum inflammation factors with 25(OH) D in the SAM algorithm required normalized data, and factors with skewed distribution were transformed in natural logarithms. Further associations between 25(OH) D and blood variables were assessed with Pearson correlation test, and if necessary, variables were logarithmically transformed to achieve linearity. Linear regression models were then applied to assess any differences between men and women in the vitamin D-associated inflammation factors that were stratified by sex. It was done by using each circulating factor of interest as the response variable and the continuous 25(OH) D values and sex as independent variables, and the interaction term of 25(OH) D and sex was entered into the model. In the CSS analysis, participants were censored with an event at the time of death from rectal cancer or with no event if dead from other causes or censored alive. Potential predictors of CSS were first analyzed in univariable Cox proportional hazard models. The vitamin D status was analyzed both by modelling continuous 25(OH) D values per 10 nmol/L of increment and categories of 25(OH) D values above or below 50 nmol/L. Because serum 25(OH) D showed no association with survival in the patients with sufficient levels (i.e.*,* increasing continuous values above the threshold of 50 nmol/L were not indicatory for improved CSS; results not shown), the final models included the categorized status. The multivariable models included covariates of deductive prognostic factors, such as ACR stage (which additionally was selected from the univariable analyses as joint expression for significant predictors) and collapsed seasons of diagnosis, and were stratified by sex. The results from all Cox proportional hazard models were expressed as hazard ratio (HR) with 95% confidence interval (CI). Differences in CSS were assessed by the log-rank test and visualized by the Kaplan-Meier method. All tests were two-sided and *p* < 0.05 was considered statistically significant.

## Results

### Baseline characteristics

Online Resource 1: Table S1 shows demographics for the study population of 129 patients (35.7% females, 64.3% males) with the expected mean age of 65 years (minimum, 41; maximum, 88). The mean serum 25(OH) D level was 68.6 nmol/L (SD, 25). No recording of vitamin D supplementation had been undertaken. The season of study inclusion was categorized as winter (1 December through 28/29 February), spring (1 March through 31 May), summer (1 June through 31 August), and fall (1 September through 30 November). The enrollment of patients was equally distributed over the year, and serum 25(OH) D did not differ significantly over the seasons. Patients presenting with T4 disease had significantly lower 25(OH) D levels than T2-T3 cases, which was reflected in the ACR advanced-stage cases

### Sex differences

**Fig. 1:**
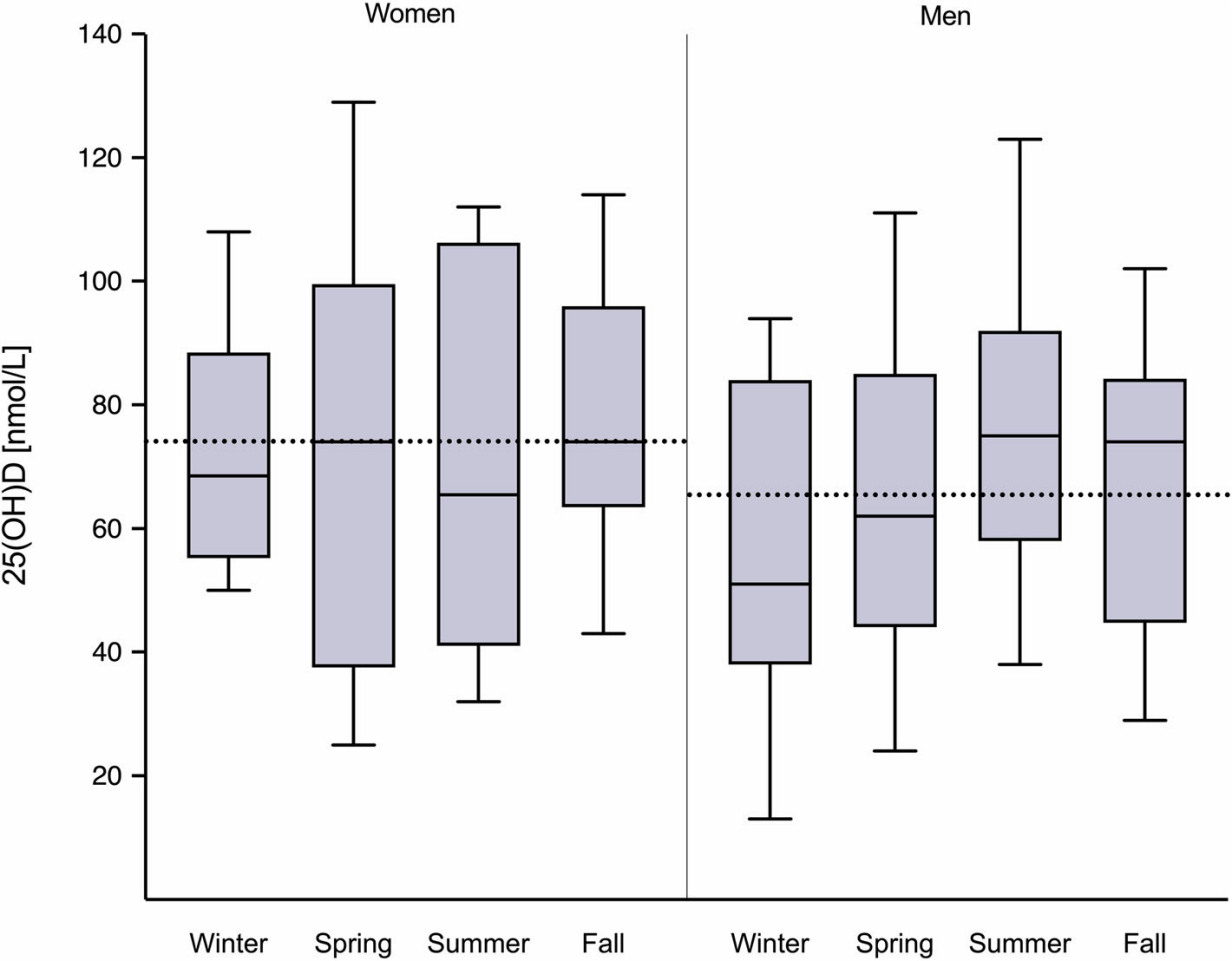
Serum 25-hydroxyvitamin D [25(OH) D] levels in women and men according to the season of rectal cancer diagnosis. Mean levels are indicated by box lines, and box upper and lower borders indicate the 25th and 75th percentiles. Minimum and maximum levels are represented by the whiskers. The dashed lines show the mean levels for the entire female and male populations

In total, 33 patients (25.6%) presented with vitamin D deficiency [circulating 25(OH) D less than 50 nmol/L], among whom men (*n* = 26; women, *n* = 7) were overrepresented (78.8%; *p* = 0.049). Accordingly, the mean 25(OH) D value was lower in men [65.5 nmol/L (SD, 24.3)] than in women [74.1 nmol/L (SD, 26.0)] (Fig. 1) but the difference was not statistically significant. For men, in contrast to the case for women, 25(OH) D at the time of rectal cancer diagnosis varied according to the season with a significant difference between the winter and summer groups, as displayed in Table 1. Because of these differences, further investigations of vitamin D status and disease features were stratified with regard to sex. Men with T4 disease had lower 25(OH) D levels than those with less extensive primary tumor. For women, involved lymph nodes (N-positive disease) were associated with lower 25(OH)D. ACR stage IV patients were lower in 25(OH) D but without being strongly statistically different from less advanced cases in women nor in men.

**Table 1:** Linear regression analyses of serum 25(OH) D and patient and disease features stratified by sex

	Women			Men		
	*n*	RC (95% CI)	*p*	*n*	RC (95% CI)	*p*
Age	46	−0.2 (−1.0, 0.6)	0.56	83	0.2 (−0.3, 0.7)	0.46
Season of inclusion						
Winter	10	Reference		19	Reference	
Spring	11	−2.2 (− 25.7, 21.3)	0.85	21	5.8 (−9.1, 20.8)	0.44
Summer	8	−1.9 (−27.4, 23.6)	0.88	21	18.4 (3.4, 33.3)	0.017
Fall	17	5.5 (−16.0, 26.9)	0.61	22	12.5 (−2.3, 27.3)	0.097
T stage						
2	11	Reference		14	Reference	
3	23	−8.6 (−27.8, 10.6)	0.37	40	−6.8 (−20.9, 7.3)	0.34
4	12	−14.9 (−36.8, 6.9)	0.18	29	−23.1 (−37.9, −8.3)	0.003
N stage						
0	21	Reference		37	Reference	
1	17	−23.5 (−39.2, 7.9)	0.004	27	3.2 (−9.1, 15.5)	0.61
2	8	−22.4 (−42.3, 2.5)	0.028	19	−4.6 (−18.3, 9.1)	0.50
M stage						
0	34	Reference		68	Reference	
1	12	−12.7 (−30.1, 4.6)	0.15	15	−6.9 (−20.6, 6.9)	0.32
ACR stage						
I	11	Reference		12	Reference	
II	11	−4.5 (−26.1, 17.0)	0.67	27	−14.3 (−30.9, 2.4)	0.091
III	12	−20.0 (−41.1, 1.1)	0.063	29	−13.5 (−30.0, 2.9)	0.11
IV	12	−21.2 (−42.3, −0.1)	0.049	15	−18.3 (−36.9, 0.3)	0.053

*25(OH) D* 25-hydroxyvitamin D, *ACR* American College of Radiology, *CI* confidence interval, *M* metastasis, *N* node, *RC* regression coefficient, *T* tumor

The sex differences associated with the vitamin D status also pertained to circulating inflammation markers. In men but not in women, common inflammation markers of advanced cancer (low hemoglobin and high values of thrombocyte count, C-reactive protein, erythrocyte sedimentation rate, and alkaline phosphatase) were inversely correlated with 25(OH) D (Table 2). Three exploratory serum factors—chemokine (C-X-C motif) ligand 7 (CXCL7; formally known as pro-platelet basic protein), interleukin-6 (IL-6), and interleukin-10—showed strong negative correlation with 25(OH) D by the SAM method (i.e.*,* very low false discovery rate, *q* = 0; Online Resource 2: Table S2) in the total study population. In contrast to the common markers, CXCL7 and IL-6 were retrieved in women but not in men in the stratified analysis (Table 2). However, when applying the interaction effect of sex and 25(OH) D value into the differences between men and women in vitamin D-associated inflammation factors, only elevated thrombocyte count, erythrocyte sedimentation rate, and alkaline phosphatase remained significant markers for men with poor vitamin D status (Online Resource 3: Table S3).

**Table 2:** Serum 25(OH) D and correlation with other circulating factors stratified by sex

	Women			Men		
	*n*	*r*^a^	*p*	*n*	*r*^a^	*p*
CEA^b^	45	−0.18	0.23	82	−0.21	0.06
CRP^b^	44	−0.09	0.55	80	−0.25	0.03
Hemoglobin	45	0.09	0.57	83	0.28	0.01
ESR	19	−0.007	0.98	35	−0.58	< 0.001
ALP	38	0.08	0.63	78	−0.32	0.004
Thrombocytes	37	0.05	0.75	76	−0.42	< 0.001
Neutrophils	34	−0.11	0.55	68	−0.20	0.09
Lymphocytes	34	0.01	0.94	68	0.21	0.08
Monocytes	34	−0.10	0.59	68	−0.22	0.07
Calcium	29	0.34	0.07	68	−0.06	0.64
Creatinine	45	−0.003	0.99	83	0.21	0.06
CXCL7	24	−0.53	0.008	39	−0.23	0.16
IL-6^b^	24	−0.49	0.015	37	−0.24	0.15
IL-10	24	−0.29	0.16	39	−0.26	0.11

*25(OH) D* 25-hydroxyvitamin D, *ALP* alkaline phosphatase, *CEA* carcinoembryonic antigen, *CRP* C-reactive protein, *CXCL7* chemokine (C-X-C motif) ligand 7, *ESR* erythrocyte sedimentation rate, *IL-6* interleukin-6, *IL-10* interleukin-10

^a^Pearson correlation coefficient

^b^Transformed in natural logarithm

### Survival

**Fig. 2:**
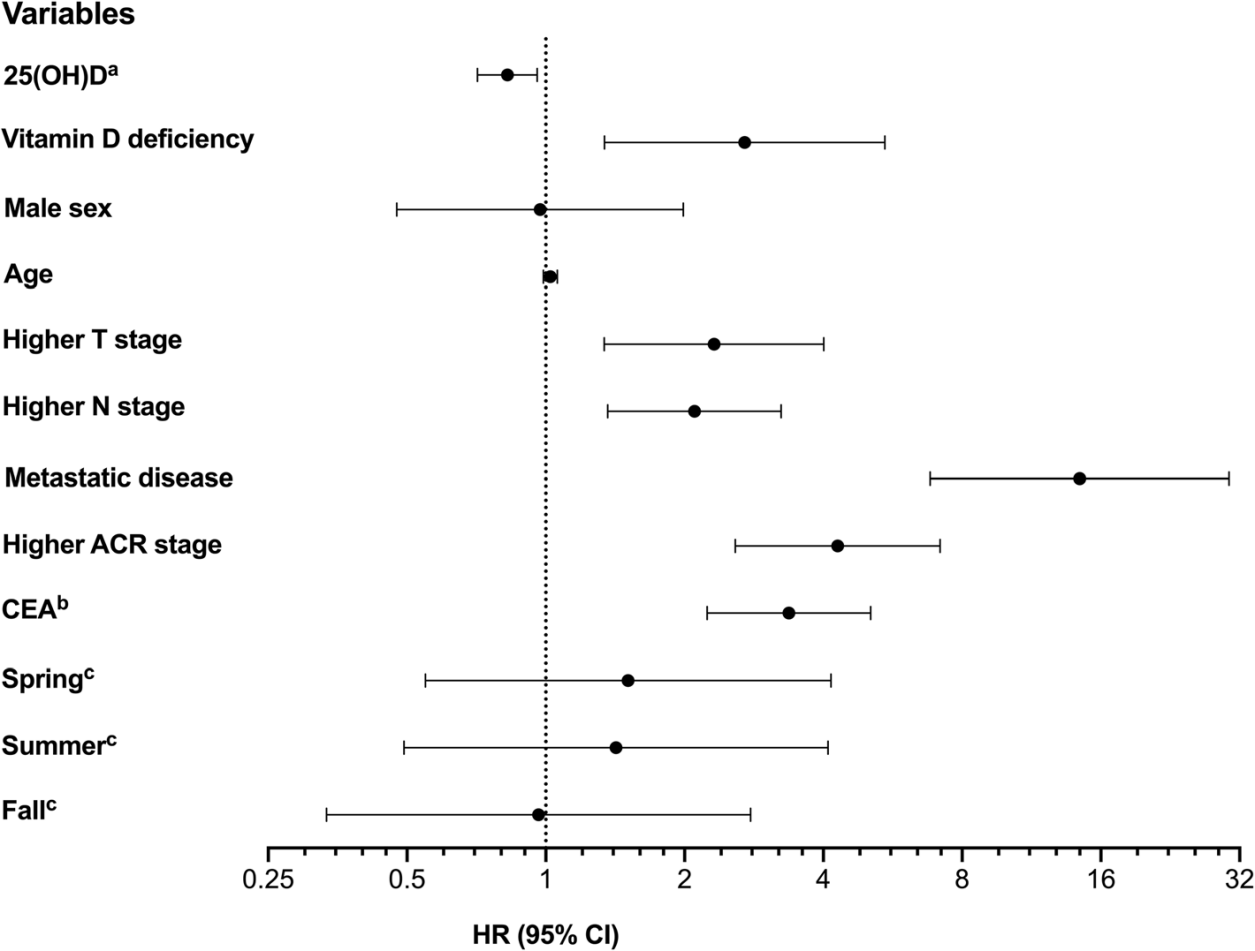
Variables associated with cancer-specific survival. Univariable hazard ratios (HR; circles) with 95% confidence intervals (CI; lines) for cancer-specific mortality; values below 1 are favorable of better survival. ^a^ Values per 10 nmol/L of increment. ^b^ Values of carcinoembryonic antigen (CEA) categorized as < 5, 5–20, and > 20 μg/L. ^c^ Winter as reference

Of the 102 study participants who presented with cancer localized within the pelvic cavity at the time of enrollment, 16 patients (15.7%) experienced metastatic progression over the follow-up period. There was no association of serum 25(OH) D with progression to metastatic disease (results not shown). Altogether, 35 patients in the total study population (27.1%) were recorded with a death event, of whom 3 individuals died from causes not related to the rectal cancer. In univariable Cox proportional hazard models in the total study population (Fig. 2), 25(OH) D levels were predictive of CSS [HR 0.83 (95% CI, 0.71–0.96) for each 10 nmol/L of increment and HR 2.70 (95% CI, 1.34–5.44) for vitamin D deficiency], as were known prognostic CRC markers such as more advanced disease (higher TNM- and ACR stages) and higher circulating levels of the tumor marker carcinoembryonic antigen [21] (categorized as < 5, 5–20, and > 20 μg/L). Due to the collinearity of these factors, only the ACR stage was included in the multivariable survival analyses, which were stratified by sex (Table 3). While 31.3% (26 of 83) of men were vitamin D-deficient, only 15.2% (7 of 46) of women belonged to this category. When analyzed by univariable models, vitamin D deficiency significantly increased HR for a CSS event in women, while for men it had borderline significant detrimental effect. In the multivariable adjusted models, deficient vitamin D status was associated with significantly enhanced risk of death for all patients [HR 5.0 (95% CI, 1.3–19.7) for women and HR 4.2 (95% CI, 1.5–11.9) for men]. Online Resource 4: Fig. S1 shows the corresponding Kaplan-Meier survival curves for the non-stratified study population.

**Table 3:** Serum 25(OH) D and CSS stratified by sex

25(OH) D, nmol/L	Women			Men		
	< 50	≥50	*p*	< 50	≥50	*p*
No. of patients	7	39		26	57	
No. of events	4	8		16	10	
Median CSS (min, max), months	27 (10, 42)	42 (3.0, 74)		34 (2.5, 68)	43 (3.0, 74)	
Univariable HR (95% CI)	5.5 (1.5, 20.1)	1	0.011	2.4 (1.0, 5.7)	1	0.053
Multivariable HR (95% CI)^a^	5.0 (1.3, 19.7)	1	0.021	4.2 (1.5, 11.9)	1	0.007

*25(OH) D* 25-hydroxyvitamin D, *CI* confidence interval, *CSS* cancer-specific survival, *HR* hazard ratio, *max* maximum, *min* minimum

^a^Adjusted for disease stage and season (winter/spring and summer/fall as collapsed categories)

## Discussion

In 129 rectal cancer patients residing at latitude 60°N and comprising the entire range of disease stages, the individuals presenting with T4 disease or the more advanced ACR stages had reduced levels of circulating 25(OH) D, with men overrepresented among vitamin D-deficient patients. Moreover, vitamin D levels varied according to the season of diagnosis among the male patients, who were also those accounting for the reduced serum 25(OH) D within the T4 category. The interconnection of low vitamin D level and systemic inflammation was distinctive for each sex as manifested by common or exploratory inflammation markers. Although vitamin D deficiency was twice as common in men, significantly higher risk of death from the malignancy was observed for the affected individuals irrespective of sex.

In our previously conducted study with neoadjuvant chemotherapy and radiation for locally advanced rectal cancer, enrolling patients from the same latitude between 2005 and 2010, serum 25(OH) D varied significantly over seasons in both sexes [13]. The seasonal sunlight variations in Norway (mainland latitude 58–71°N) are significant [22]. In 2013, the Nordic Nutrition Recommendations experts recommended vitamin D supplementation in wintertime for the general population [16]. A tremendous interest in possible health effects of vitamin D beyond its regulation of the bone metabolism and a considerable increase of users of prescription-only vitamin D supplements and over-the-counter dietary supplements have been observed over the past decade [23]. As the present population-based study, enrolling rectal cancer patients between 2013 and 2017, demonstrated seasonal variation in circulating 25(OH) D only in men, one can speculate that women might have changed habits towards better vitamin D status over the entire calendar year. In the general population of Denmark (latitude 55–57°N) in 2012–2014, women had higher circulating 25(OH) D than men and were supplement users to a greater extent [24]. Observational studies and meta-analyses on populations from the wider European territories and in North America, Japan, and Australia have shown that high vitamin D level may lower CRC risk [1, 2, 25, 26] and improve prognosis [3, 4, 27–30]. A prospective study in the United States in patients with metastatic CRC on first-line systemic therapy revealed that those randomized to high-dose vitamin D supplementation had better outcome than the control patient group given a standard vitamin D dose [31].

Consistent with the findings in our previous study [13], the present study showed that rectal cancer patients with T4 disease and poor outcome had significantly reduced 25(OH) D levels at the time of diagnosis. The study population exhibited lower 25(OH) D values by each step of more advanced T stage, with the significant association between low 25(OH) D and T4 driven by the male participants. In contrast, low 25(OH) D values were associated with N-positive disease in the female participants. Each of these interconnected factors were predictive of shortened CSS. Although deficient vitamin D status was much less common in women than in men, it was strongly associated with enhanced risk of death from the rectal cancer for both sexes as assessed by the multivariable survival models. The diagnostic high-resolution magnetic resonance imaging of the rectum identifies common surgical and pathological risk factors [32–34]. Yet, our results request raised awareness of sex disparities and consequential risk factors—such as vitamin D deficiency—beyond those related to the disease manifestations within the pelvic cavity to further improve the accuracy of patient stratification to therapy.

A unique discovery from our investigations was that sex differences associated with the vitamin D status also pertained to the modes of systemic inflammation. The clinical syndrome of SIR, defined by elevated circulating white blood cell- and thrombocyte counts and acute-phase proteins such as C-reactive protein [35], has been consistently observed to confer poor outcome [36], as shown valid in all stages of CRC [8, 9]. In the present study population, the classic SIR was related to low vitamin D in the male patients, also when the associations were compared by entering sex and 25(OH) D level as interacting factors in the statistical model. In the female patients, low vitamin D correlated with elevated IL-6 and CXCL7 in the circulation. In CRC, IL-6 links tumor necrosis to SIR [37]. Clinically, IL-6 has widespread organ effects reflected by the typical SIR markers [38]. Excessive or persistent IL-6 production is a driving feature in the malignant progression [39, 40]. It has long been known that vitamin D suppresses IL-6 production by experimental immune cell models [41–43]. After vascular injury, CXCL7 is released from thrombocytes to promote neoangiogenesis [44]. Vitamin D represses the transcription of several factors linked to tumor angiogenesis [45]. Interestingly, CXCL7 has been proposed as a circulating biomarker for CRC detection [46]. With regard to vascular processes, we recently showed that hemodynamic factors of the rectal cancer differ and result in distinctive outcomes in men and women [47, 48]. In our previous vitamin D study, a weak inverse correlation between circulating 25(OH) D and SIR markers appeared in all patients analyzed together [13]—it might be that stronger and more distinct modes of systemic inflammation with respect to the vitamin D status had been revealed if male and female patients had been analyzed separately.

This study has weaknesses. The sample size was limited and results must be interpreted cautiously. On the other hand, given the unselected study recruitment at a hospital with demographics representative for the entire nation, the cohort can be considered to reflect the full landscape of rectal cancer. Secondly, the reported set of analyses was not planned at the time of patient enrollment and thus, results are subject to confounding effects. Specifically, the assessed vitamin D levels might be a surrogate for other modifiable lifestyle factors associated with rectal cancer presentation and outcome; however, potential effects of sunlight exposure (other than the production of vitamin D) on CSS were adjusted for. Finally, the use of multiplex technology for assessing circulating factors comes with certain limitations. The accuracy and precision of such panel screens depend on the selection of antibodies and the standardization of the measurements.

## Conclusions

The results from the present examinations further substantiate the positive impact of a sufficient vitamin D status on the presentation and outcome of rectal cancer. Specifically, the data opens for an interesting theory that insufficient vitamin D levels may operate distinctive patterns of adverse systemic inflammation in men and women, adding sex-related aspects to be considered in future investigations into vitamin D and CRC.

## Supplementary Information

**Additional file 1: Table S1.** Patient and disease features and serum 25(OH)D in the total study population.

**Additional file 2: Table S2.** Results from multiple correlation analysis between serum 25-hydroxyvitamin D and inflammation proteins using the Significance Analysis for Microarrays (SAM) method.

**Additional file 3: Table S3.** Linear regression analyses of the interaction effect of serum 25(OH)D and sex on differences between men and women in vitamin D-associated systemic inflammation factors.

**Additional file 4: Figure S1.** The difference in cancer-specific survival (CSS) for patients with sufficient (≥50 nmol/L) and deficient (< 50 nmol/L) serum 25-hydroxyvitamin D (*p* = 0.004; by log-rank test).

## Data Availability

The datasets generated and/or analyzed during the current study are available from the corresponding author on reasonable request.

## Abbreviations

25(OH) D: 25-hydroxyvitamin D
ACR: American College of Radiology
CI: Confidence interval
CRC: Colorectal cancer
CSS: Cancer-specific survival
CXCL7: Chemokine (C-X-C motif) ligand 7
HR: Hazard ratio
IL-6: Interleukin-6
SAM: Significance Analysis for Microarrays
SD: Standard deviation
SIR: Systemic inflammatory response
