# Supplementary material for: Sex disparities in vitamin D status and the impact on systemic inflammation and survival in rectal cancer
Source: BMC Cancer. 2021 May 11;21:535. doi: 10.1186/s12885-021-08260-2 (PMC8111928; doi:10.1186/s12885-021-08260-2)
Supplement: Supplementary file 1 — Additional file 1: Table S1. Patient and disease features and serum 25(OH)D in the total study population. [file 12885_2021_8260_MOESM1_ESM.docx]

**Table S1** Patient and disease features and serum 25(OH)D in the total study population

|  |  | *n (%)* | Mean 25(OH)D (SD), nmol/L | *r*^a^ | *p* |
| --- | --- | --- | --- | --- | --- |
| Mean age (SD), years | 65.0 (10.4) | 129 |  | 0.01 | 0.91 |
| Mean 25(OH)D (SD), nmol/L | 68.6 (25.1) | 129 |  |  |  |
| Sex | Women | 46 (35.7) | 74.1 (26.0) |  |  |
|  | Men | 83 (64.3) | 65.5 (24.3) |  | 0.063 |
| Season of inclusion | Winter | 29 (22.5) | 61.9 (24.6) |  |  |
|  | Spring | 32 (24.8) | 64.9 (27.8) |  |  |
|  | Summer | 29 (22.5) | 73.5 (24.8) |  |  |
|  | Fall | 39 (30.2) | 72.8 (22.7) |  | 0.17 |
| T stage | 2 | 25 (19.4) | 79.2 (23.7) |  |  |
|  | 3 | 63 (48.8) | 71.3 (22.6) |  |  |
|  | 4 | 41 (31.8) | 57.7 (26.2) |  | 0.001 |
| N stage | 0 | 58 (45.0) | 73.2 (26.1) |  |  |
|  | 1 | 44 (34.1) | 66.6 (24.9) |  |  |
|  | 2 | 27 (20.9) | 61.9 (22.2) |  | 0.13 |
| M stage | 0 | 102 (79.1) | 70.3 (25.9) |  |  |
|  | 1 | 27 (20.9) | 62.0 (21.1) |  | 0.13 |
| ACR stage | I | 23 (17.8) | 81.9 (24.5) |  |  |
|  | II | 38 (29.5) | 68.9 (24.8) |  |  |
|  | III | 41 (31.8) | 65.0 (26.2) |  |  |
|  | IV | 27 (20.9) | 62.0 (21.1) |  | 0.03 |

^a^ Pearson correlation coefficient

*25(OH)D* 25-hydroxyvitamin D, *ACR* American College of Radiology, *M* metastasis, *N* node, *SD* standard deviation, *T* tumor
