# Supplementary material for: Sex disparities in vitamin D status and the impact on systemic inflammation and survival in rectal cancer
Source: BMC Cancer. 2021 May 11;21:535. doi: 10.1186/s12885-021-08260-2 (PMC8111928; doi:10.1186/s12885-021-08260-2)
Supplement: Supplementary file 2 — Additional file 2: Table S2. Results from multiple correlation analysis between serum 25-hydroxyvitamin D and inflammation proteins using the Significance Analysis for Microarrays (SAM) method. [file 12885_2021_8260_MOESM2_ESM.docx]

**Table S2** Results from multiple correlation analysis between serum 25-hydroxyvitamin D and inflammation proteins using the Significance Analysis for Microarrays (SAM) method

| Negative correlations | | |  | Positive correlations | | |
| --- | --- | --- | --- | --- | --- | --- |
| Protein | *d* | *q* |  | Protein | *d* | *q* |
| CXCL7 | –2.99 | 0 |  | VEGF | 2.39 | 39.5 |
| IL-6 | –2.45 | 0 |  | FAS | 1.95 | 55.7 |
| IL-10 | –2.23 | 0 |  | RAGE | 1.63 | 64.3 |
| GRO-b | –1.83 | 13.5 |  | TPO | 1.24 | 72.4 |
| PDGFDD | –1.79 | 13.5 |  | ERBB2 | 1.23 | 72.4 |
| TARC | –1.68 | 13.5 |  | NCAM1 | 1.21 | 72.4 |
| IL-17 | –1.62 | 13.5 |  | CD31 | 1.15 | 72.4 |
| CCL5 | –1.33 | 37.9 |  | TNF-R-II | 1.11 | 72.4 |
| PDGFCC | –1.32 | 37.9 |  | Tie1 | 0.88 | 72.4 |
| PDGFBB | –1.25 | 37.9 |  | LCN2 | 0.78 | 72.4 |
| TNF-a | –1.12 | 39.5 |  | CXCL6 | 0.76 | 72.4 |
| SPARC | –1.05 | 39.5 |  | RANKL | 0.73 | 72.4 |
| P-selectin | –0.84 | 55.7 |  | Tie2 | 0.58 | 89.4 |
| CXCL8 | –0.82 | 55.7 |  | Tie1 | 0.54 | 89.4 |
| S100A8 | –0.80 | 55.7 |  | IFN-g | 0.49 | 89.4 |
| CXCL13 | –0.68 | 55.7 |  | L-selectin | 0.46 | 89.4 |
| Osteopontin | –0.67 | 55.7 |  | CX3CL1 | 0.42 | 89.4 |
| CD40 | –0.66 | 55.7 |  | CXCL14 | 0.42 | 89.4 |
| ANGPT1 | –0.53 | 63.2 |  | CD27 | 0.36 | 89.4 |
| SDF1-a | –0.50 | 63.2 |  | OPG | 0.35 | 89.4 |
| CXCL4 | –0.49 | 63.2 |  | TRAIL | 0.31 | 89.4 |
| E-selectin | –0.46 | 63.2 |  | PDGFAA | 0.23 | 89.4 |
| CCL13 | –0.43 | 63.2 |  | GP130 | 0.22 | 89.4 |
| TNF-R-I | –0.43 | 63.2 |  | CXCL16 | 0.21 | 89.4 |
| Angiogenin | –0.40 | 63.2 |  | IL-5 | 0.16 | 89.4 |
| VCAM1 | –0.27 | 73.5 |  | FLT3LG | 0.15 | 89.4 |
| IL-1ra | –0.12 | 81.2 |  | CD40L | 0.11 | 89.5 |
| IL-4 | –0.04 | 91.3 |  | CCL18 | 2.39 | 89.5 |

The SAM score, *d*, is the statistic strength and the *q* value the lowest false discovery rate at which the protein is called significant
