# Supplementary material for: Sex disparities in vitamin D status and the impact on systemic inflammation and survival in rectal cancer
Source: BMC Cancer. 2021 May 11;21:535. doi: 10.1186/s12885-021-08260-2 (PMC8111928; doi:10.1186/s12885-021-08260-2)
Supplement: Supplementary file 3 — Additional file 3: Table S3. Linear regression analyses of the interaction effect of serum 25(OH)D and sex on differences between men and women in vitamin D-associated systemic inflammation factors. [file 12885_2021_8260_MOESM3_ESM.docx]

**Table S3** Linear regression analyses of the interaction effect of serum 25(OH)D and sex on differences between men and women in vitamin D-associated systemic inflammation factors

|  | 25(OH)D  (95% CI) | Sex^a^  (95% CI) | Interaction term  (95% CI) | *p* |
| --- | --- | --- | --- | --- |
| CRP | –0.005  (–0.02, 0.01) | 0.67  (–0.81, 2.1) | –0.01  (–0.03, 0.01) | 0.38 |
| Hemoglobin | 0.004  (–0.1, 0.02) | –0.56  (–2.3, 1.2) | 0.02  (–0.01, 0.04) | 0.15 |
| ESR | –0.003  (–0.27, 0.26) | 26.0  (3.10, 48.9) | –0.37  (–0.69, 0.05) | 0.026 |
| ALP | 0.08  (–0.4, 0.6) | 54.8  (9.40, 100) | –0.70  (–1.3, 0.09) | 0.026 |
| Thrombocytes | 0.14  (–0.95, 1.2) | 101  (–1.83, 205) | –1.85  (–3.20, 0.50) | 0.008 |
| CXCL7 | –106  (–181, 30.8) | –4664.5  (–13251, 3922.2) | 43.6  (–70.2, 158) | 0.45 |
| IL-6 | –0.02  (–0.04, 0.01) | –0.22  (–2.2, 1.8) | 0.01  (–0.02, 0.03) | 0.71 |

^a^ Female as reference

*25(OH)D* 25-hydroxyvitamin D, *ALP* alkaline phosphatase, *CI* confidence interval, *CRP* C-reactive protein, *CXCL7* chemokine (C-X-C motif) ligand 7, *ESR* erythrocyte sedimentation rate, *IL-6* interleukin-6
