# Supplementary material for: Sex disparities in vitamin D status and the impact on systemic inflammation and survival in rectal cancer
Source: BMC Cancer. 2021 May 11;21:535. doi: 10.1186/s12885-021-08260-2 (PMC8111928; doi:10.1186/s12885-021-08260-2)
Supplement: Supplementary file 4 — Additional file 4: Figure S1. The difference in cancer-specific survival (CSS) for patients with sufficient (≥50 nmol/L) and deficient (< 50 nmol/L) serum 25-hydroxyvitamin D (p = 0.004; by log-rank test). [file 12885_2021_8260_MOESM4_ESM.docx]

**Fig. S1** The difference in cancer-specific survival (CSS) for patients with sufficient (≥50 nmol/L) and deficient (<50 nmol/L) serum 25-hydroxyvitamin D (*p* = 0.004; by log-rank test)
